# Sensitive detection and apoptotic cell death induction of adult T-cell leukemia/lymphoma (ATL) cells with photodynamic actions

**DOI:** 10.1186/1742-4690-11-S1-P5

**Published:** 2014-01-07

**Authors:** Takashi Oka, Hirofumi Fujita, Lamia Abd Al-Kader, Ichiro Murakami, Atae Utsunomiya, Tadashi Yoshino

**Affiliations:** 1 Department of Pathology, Graduate School of Medicine, Dentistry and Pharmaceutical Sciences, Okayama University, Okayama, Japan; 2 Department of Cytology and Histology, Graduate School of Medicine, Dentistry and Pharmaceutical Sciences, Okayama University, Okayama, Japan; 3 Department of Molecular Pathology, Tottori University Medical School, Yonago, Japan; 4 Department of Hematology, Imamura Bun-in Hospital, Kagoshima, Japan

## 

Adult T cell leukemia/lymphoma (ATL) is an aggressive malignant disease of CD4 positive T lymphocytes caused by infection with human T cell leukemia virus type I (HTLV-I). HTLV-1 causes ATL in 3-5% of infected individuals after a long latent period of 40 to 60 years. The acute and lymphoma types are aggressive ATL characterized by resistance to chemotherapy and a poor prognosis. Leukemia/lymphoma cells and rapidly proliferating cells preferentially accumulate endogenous photosensitizer protoporphyrin IX (PpIX) when supplemented with 5-aminolevulinic acid (ALA). Treatment with 1mM ALA for 48h induced 10 to 100 times accumulation of PpIX in ATL leukemic cell lines and HTLV-I (+) T cell lines than that in healthy PBMCs. Specific induction of apoptosis was observed after 10 min light exposure (28 mW/cm^2^) using Na-Li lamp in ATL leukemic cell lines and HTLV-I (+) T cell lines. ATL patient PBMC specimen showed strong accumulation of PpIX with the treatment of ALA compared to the healthy donor and HTLV-I carrier PBMCs, which could be useful for the diagnostic purposes and monitoring the patient status with high sensitivity. Photodynamic therapy is potentially hopeful treatment especially for lymphoma type ATL as a relatively selective, minimal or no scarring, non-invasive, safe, simultaneous and repeatable multiple lesions treatable modality.

